# Anti-PD-1 antibody enhances homeostatic proliferation–induced antitumor immunity during lymphopenia recovery

**DOI:** 10.3389/fimmu.2025.1563894

**Published:** 2025-05-02

**Authors:** Zike Yang, Ying Zhu, Qian Wang, Qing Lin, Yahui Liu

**Affiliations:** ^1^ Department of Oncology, Zhongshan Hospital of Xiamen University, School of Medicine, Xiamen University, Xiamen, Fujian, China; ^2^ Department of Rehabilitation, Zhongshan Hospital of Xiamen University, School of Medicine, Xiamen University, Xiamen, Fujian, China; ^3^ Department of Oncology, Southern Medical University Hospital of Integrated Traditional Chinese and Western Medicine, Southern Medical University, Guangzhou, China; ^4^ Department of Obstetrics and Gynecology, The Third Affiliated Hospital/The Third Clinical Medicine School of Southern Medical University, Guangzhou, China

**Keywords:** PD-1/PD-L1, lymphopenia, immunotherapy, melanoma, homeostatic proliferation, antitumor immunity

## Abstract

**Introduction:**

Lymphopenia induced by radiotherapy or chemotherapy can promote homeostatic proliferation of residual or adoptive lymphocytes, potentially enhancing antitumor immunity. However, this immunity diminishes rapidly with tumor progression, and the underlying mechanisms remain unclear. This study investigates the role of PD-1 signaling in homeostatic proliferation–induced antitumor immunity in malignant melanoma.

**Methods:**

We evaluated T-cell dynamics in lymphopenic mice, analyzing PD-1 expression, IFN-γ production by CD8^+^ T cells, and T-cell cytotoxicity during homeostatic proliferation. The PD-1/PD-L1 axis was blocked using anti-PD-1 antibodies to assess its impact on T-cell function, dendritic cell (DC) activation, and memory T-cell differentiation.

**Results:**

Although T cells proliferated continuously in lymphopenic mice, IFN-γ+ CD8^+^ T cells declined over time. PD-1 expression on T cells increased progressively and correlated negatively with effector T-cell cytotoxicity. PD-1 blockade enhanced the recognition of tumor-associated antigens (TAAs) by homeostatically proliferating (HP) T cells, activated DCs, and increased IFN-γ+ CD8+ T-cell numbers. Additionally, it boosted T-cell cytotoxicity and promoted the conversion of tumor-specific effector T cells into central memory T cells.

**Discussion:**

These findings indicate that the PD-1/PD-L1 axis plays a critical role in immune tolerance during homeostatic proliferation. Anti-PD-1 therapy may enhance antitumor immunity during lymphopenia recovery after chemotherapy or radiotherapy, offering a potential strategy to sustain T-cell–mediated tumor control.

## Introduction

Under normal circumstances, the immune system could recognize and remove tumor cells in the tumor microenvironment. However, tumor cells adopt different strategies to inhibit the immune system and cannot be killed by immune cells, so that they could survive in all stages of anti-tumor immune response. The goal of cancer immunotherapy is to induce a sustained antitumor immune response through the *in vivo* generation of functional tumor antigen-specific T cells that are not restrained by normal or cancer-induced tolerance mechanisms (1).

The homeostasis of T cells refers to the relative constant number of T cell populations at the overall level. If T cells were significantly reduced for some reason, the remaining T cells in the periphery will proliferate spontaneously to restore the normal size of T cells population and maintain homeostasis (2). In the condition of sharp decrease of T cells, T cells proliferated steadily through the action of self-peptide-MHC complex presented by dendritic cells(DCs) to restore the original T cell pool size. The proliferated T cells have remarkable self-responsiveness and function as both effector and memory T cells (3). Tumor associated antigen were usually poor target when lymphocytes were sufficient, because they were often self-antigens and had weak immunogenicity. Lymphopenic condition could create an environment to induce an enhanced antitumor immunity through the homeostatic proliferation of residual or adoptive lymphocytes as shown in lymphopenic animal models (4, 5). However, the antitumor immunity seemed to decrease rapidly with the growth of tumor in the later stage of homeostatic proliferation, and the mechanism remained elusive (6).

The Programmed death-1 (PD-1) was a key regulator of T cell function and fate. It was up-regulated after T cell activation, but was considered to be a sign of T cell exhaustion, which was a kind of cell state with low function found in tumor infiltrating lymphocytes of patients with advanced cancer (7, 8). It was worth noting that the expression of PD-1 ligand PD-L1 and PD-L2 in a variety of tumors was related to poor prognosis. The anti-PD-1/PD-L1 blocking antibody had shown effective anti-tumor immune response and promising results in a variety of cancer patients (9–11).

PD-1/PD-L1 axis was not only involved in tumor immune escape, but also closely related to chronic infection, transplant rejection and autoimmune diseases (12, 13). Notably, recent studies have demonstrated that genetic disruption of PD-1 in T cells using CRISPR/Cas9 technology enhances antitumor immunity in lymphopenic conditions. For instance, adoptive transfer of PD-1 knockout T cells into lymphopenic mice after sublethal irradiation significantly inhibited tumor growth and promoted T cell homeostatic proliferation, suggesting synergistic effects between lymphopenia and PD-1 blockade (14). Additionally, PD-1 has been shown to regulate tonic signaling and lymphopenia-induced T cell proliferation, further underscoring its role in immune tolerance during lymphopenia (15). However, the mechanism of PD-1/PD-L1 axis on antitumor immunity induced by homeostatic proliferation of T cells in lymphopenic condition remained elusive. In this study, we first examined the dynamic change of PD-1 expression on T cells and analyzed the connection between PD-1 expression and the exhaustion of cytotoxic T lymphocytes (CTL) when the number of T cells recovered from lymphopenic condition. Anti-PD-1 antibodies were used in this study to evaluate the effects of PD-1/PD-L1 blockade on homeostatic proliferation–driven antitumor immunity. We further analyzed the role and mechanism of PD1/PD-L1 signaling on the antigen recognition, differentiation, activation, killing, survival and prognosis of homeostasis proliferating T cells. This study was not only important for elucidating the mechanism of the wane of homeostatic proliferation–driven antitumor responses, but also provided a new theoretical basis for the combined treatment of anti-PD-1 therapy during recovery from lymphopenic condition after chemotherapy or radiotherapy.

## Materials and methods

### Animal models of homeostatic proliferation

Female C57BL/6 mice (6- to 8-week-old) were purchased from Laboratory Animal Center of Southern Medical University (Guangzhou, China), and were housed under sterilized condition. Animal studies were conducted in accordance with the institutional guidelines and approved by the Animal Care and Use Committee of Southern Medical University, China. C57BL/6 mice received a sub-lethal (650 cGy) dose of total body irradiation on the day before immune cells infusion. The irradiated mice received intravenous infusion of 2×10^6^ splenic T cells. As a control, nonirradiated mice were untreated (control group) or received T lymphocyte infusion (T cell infusion group).

### Tumor challenge and measurement

Murine malignant melanoma cell line B16 were purchased from China Center for Type Culture Collection. B16 cells were resuspended in PBS at a concentration of 5×10^6^ cells/mL. Each mouse was injected s.c. laterally with a volume of 0.1ml (5×10^5^ tumor cells). The size of the tumor was measured by a caliper, and the average value was determined by taking the maximum diameter of the tumor and its perpendicularity. Tumor volume was calculated by (length×width^2^)/2. Euthanasia was carried out in mice with tumor length greater than 20 mm.

### ELISpot assays

IFN-γ ELISpot kits (BD Biosciences, USA) were used according to the manufacturer’s instructions. Briefly, 30Gy-irradiated B16 or 4T1 (5×10^4^) cells and T cells (1×10^5^) were cocultured in 96-well plates precoated with mouse IFN-γ (BD Biosciences, USA) at 37°C for 20h in complete RPMI medium in triplicate. After the cell suspension was rinsed with deionized water, biotinylated anti mouse IFN-γ antibody (2μg/ml) was added and incubated at room temperature for 2 hours. After extensive washing, streptavidin horseradish peroxidase solution was added and incubated at room temperature for 1 hour. After washing, an aminoethyl carbozole substrate solution was added and incubated for 15 min. Spots were counted under a stereomicroscope after washing the plate.

### *In vivo* depletion of T and NK cells

To deplete specific immune effector cell subsets before and during chemotherapy or radiotherapy, the transplanted mice received intravenous injection of 0.3 mg monoclonal antibody of anti-CD4^+^ (clone GK1.5, rat IgG2b, Bioxcell, USA) or anti CD8^+^ (clone Lyt-2.1, mouse IgG2b, Bioxcell, USA) or 0.5 mg of antiasialo GM1 antibody (Wako Pure Chemical Industries, Ltd). The intravenous injection started on the inoculation day of B16 cells, and was repeated every 5 to 6 days throughout the experiment to ensure the depletion of target cell types.

### CFSE labeling

Purified T cells were resuspended in cold PBS at a concentration of 2×10^7^ cells/ml. An equal volume of PBS containing 5 mmol/L carboxyfluorescein diacetate, succinimidyl ester (CFSE) labeled with FITC was added, and cells were incubated at room temperature for 6 min. The cells were washed with an excess of cold FCS (Thermo Fisher Scientific, Waltham, MA, USA) and then washed three times with a cold DMEM containing 10% FCS. As mentioned above, CFSE labeled T cells were injected intravenously and flow cytometry was used to analyze spleen cells or lymph node cells at a specified time point.

### Flow cytometry

At the indicated time points, tumor draining lymph nodes(tumor-DLNs) and spleens were harvested from the mice, and minced into small fragments and mechanically dispersed in 3-5 ml cold PBS. After filtering with 70μm cell strainer (BD Falcon, USA), single-cell suspensions were adjusted to 1×10^6^ cells in 100μl of PBS. After this, single-cell suspensions of tumor cells were stained for 30min at 4-8°C with 1μg of antibodies or CFSE labeled with FITC, PE, APC or PerCP fluorochromes. CD45, CD3, CD4, CD8, PD-1, IFN-γ, BCL-2, Annexin-V, Gr-1, CD11b, CD44, CD62L, CD122 or CD127 antibodies and matched isotypic control antibodies(BD Biosciences, USA) labeled with fluorochromes were used for flow cytometry in the study. Cells were washed twice by cold PBS to clean the unbound antibody and analyzed by FACSCalibur (BD Biosciences, USA). Irrelevant IgG mAbs were used as a negative control. Ten thousand live events were acquired for analysis.

### Statistical analysis

All data were expressed as means ± SEM. Results of tumor volume and flow cytometry were assessed using one way ANOVA. Differences were considered to be statistically significant when *P<0.05*. All statistical analyses were performed using SPSS 20.0 (IBM, Armonk, NY, USA).

## Results

### Effects of sublethal radiotherapy on homeostatic proliferation of T cells

**Figure 1:**
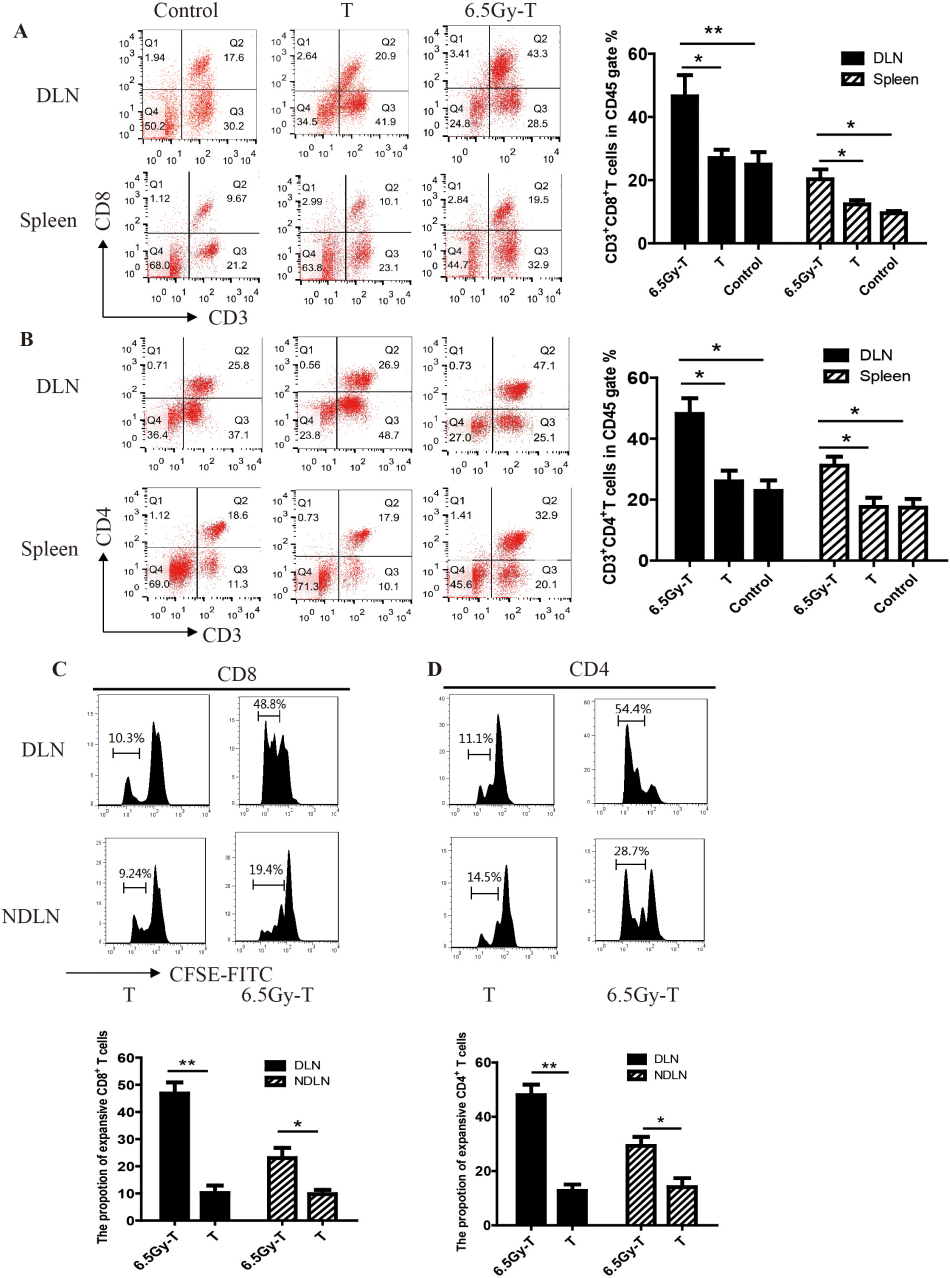
The homeostatic proliferation of T cells in lymphopenic mice. A-B. Flow cytometry analysis of CD3^+^ CD8^+^ T lymphocytes **(A)** and CD3^+^CD4^+^ T lymphocytes **(B)** in CD45^+^ gate in tumor-DLNs and spleen(n=5). C-D. CFSE dilution of CD4^+^ and CD8^+^ T cells in tumor-DLNs or tumor-NDLNs. Splenic T cells labeled with CFSE were infused to lymphopenic mice after sublethal irradiation(n=5). CD8^+^ **(C)** and CD4^+^ **(D)** T cells were isolated to analyze the CFSE dilution by flow cytometry after 10 days. (n=5) *P<0.05. **P<0.01.

To investigate the effect of combined treatment of sublethal irradiation and immune cells infusion on local antitumor immunity and system immunity, we analyzed the immune status in the tumor microenvironment and spleen after combined treatment in C57BL/6 mice. Flow cytometry analysis revealed a significant increase in the percentage of CD3^+^CD8^+^ and CD3^+^CD4^+^ T cells in tumor-DLNs and spleen in combined treatment mice (**Figures 1A, B**). To investigate whether the increased number of transferred T cells in spleen and lymph nodes of lymphopenic mice were due to cell proliferation, transferred T cells were labeled with CFSE and analyzed for CFSE dilution by flow cytometry in lymphopenic mice. As shown in **Figures 1C, D**, transferred CD4^+^ and CD8^+^ T cells underwent several rounds of proliferation in tumor-DLNs and tumor-non draining lymph nodes (tumor-NDLNs) on day 10 after infusion into sublethally irradiated mice (**Figures 1C, D**). These data suggested that combined treatment of sublethal irradiation and immune cells infusion could effectively enhance local antitumor immunity while expand system immunity.

### Despite ongoing T cells proliferation in tumor-DLNs of lymphopenic mice, functional tumor antigen–specific CD8^+^ T cells wane over time

**Figure 2:**
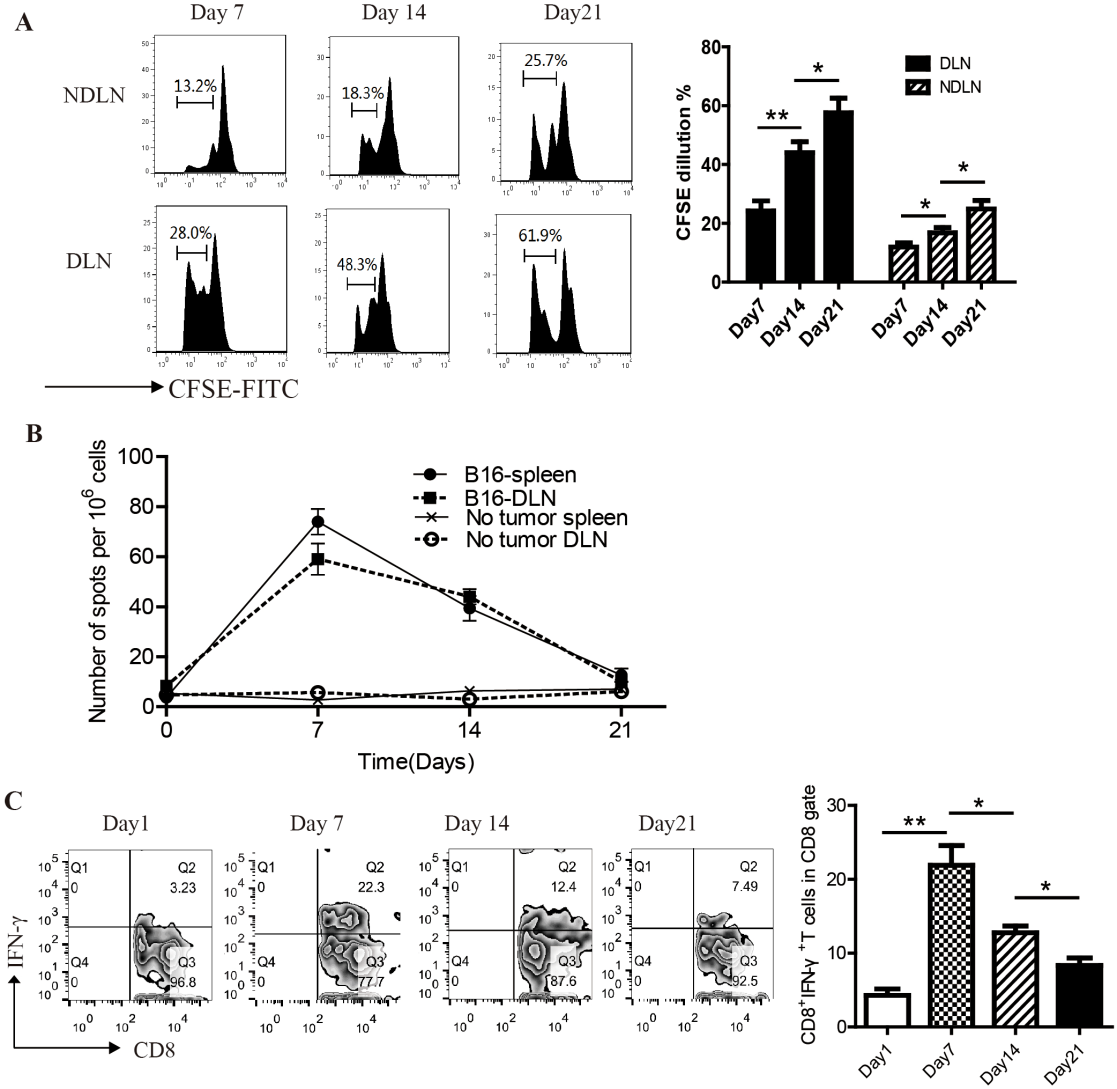
Functional tumor antigen–specific CD8+ T cells wane over time in lymphopenic mice. **(A)** CFSE dilution of CD8^+^ T cells in tumor-DLNs and NDLNs at the time point of Day7, 14 and 21(n=5). **(B)** Tumor-DLNs were harvested from tumor-bearing mice at the indicated time points, and DLN cells were restimulated *in vitro* with the irradiated B16 cell and assayed by ELISpot for evidence of function as indicated by IFN-γ production(n=5).**(C)** Flow cytometry analysis of IFN-γ-releasing CD8^+^T cells in tumor-DLNs on day7, 14, 21(n=5). *P<0.05. **P<0.01.

In an attempt to understand the relationship between antitumor immunity and the homeostatic proliferation of T cells, tumor-draining and non-draining lymph node were harvested on days 7, 14, and 21 and analyzed for CFSE dilution by flow cytometry. On day 7, there was about 28% CFSE dilution of T cells in the tumor-DLNs, indicating spontaneous antigen presentation. On day 14, as the tumor size increased over time, much greater CFSE dilution (48.3%) was present in the tumor-DLNs. On day 21, the majority of T cells (61.9%) showed evidence of proliferation (**Figure 2A**).

At the same time, we analyzed the effector function of tumor-specific T cells on days 7, 14, and 21 using flow cytometry and IFN-γ ELISpot. On day 7, DLN cells from tumor-challenged mice showed a substantial number of IFN-γ-producing cells in response to irradiated B16 cells. However, the frequency of functional T cells diminished over time, such that by day 21 only background numbers of IFN-γspots were observed (**Figure 2B**). Consistent results were obtained in flow cytometry analysis of IFN-γ-releasing CD8^+^ T cells (**Figure 2C**). These results suggested that although T cells continued proliferate in tumor-DLNs of lymphopenic mice, functional tumor antigen–specific CD8^+^ T cells wane over time.

### *In vivo* T cell hyporesponsiveness in later stage of T cell homeostatic proliferation were attributed to the upregulation of PD-1 expression

**Figure 3:**
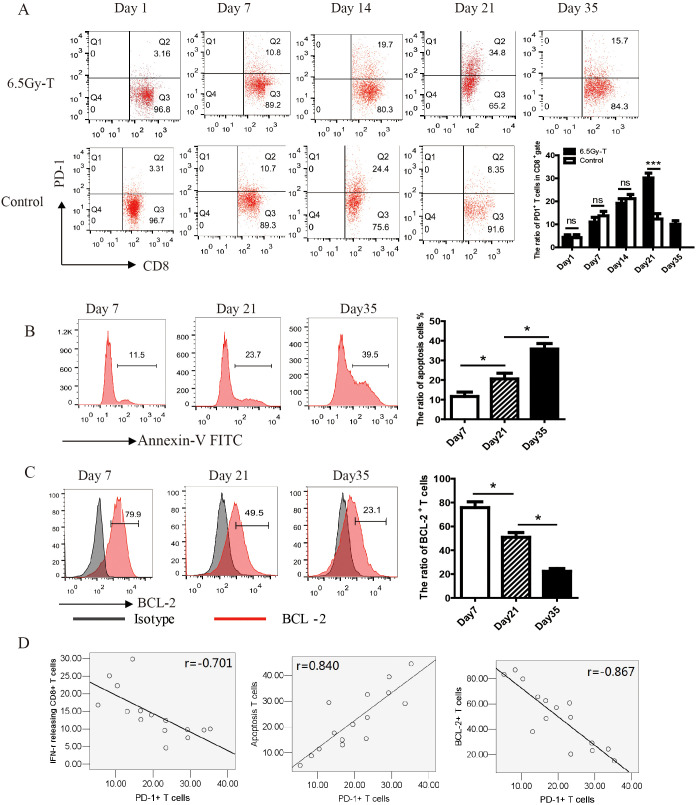
*In vivo* T cell hyporesponsiveness in later stage of T cell homeostatic proliferation were attributed to the upregulation of PD-1 expression. **(A)** The kinetics of PD-1 expression on CD8^+^ T cells during reconstitution of the lymphopenic environment(n=5). **(B, C)**. The ratio of apoptosis cells **(B)** and BCL-2^+^ T cell **(C)** in CD8^+^ T cells at the time point of day 7, 21 and 35(n=5). **(D)** The correlation analysis showed that PD-1 expression on CD8^+^ T cells was significantly negatively related to the ratio of IFN-γ-releasing CTL (n=15, P=0.025) and the expression of BCL-2 on CD8^+^ T cells (n=15, P=0.009), but positively related to the ratio of apoptosis T cells (n=15, P=0.012) *P<0.05. ns: not significant, p>0.05.

In order to understand the mechanism of T cell hyporesponsiveness in later stage of T cell homeostatic proliferation, we conducted time course experiments to determine the dynamic change of PD-1 expression on CD8^+^ T cells during recovery from lymphopenic condition. Barely detectable before transfer, PD-1 expression slightly increased at day 7, peaked at day 21, and returned to low levels by day 35. (**Figure 3A**). On day 21, PD-1 expression on CD8^+^ T cells in lymphopenic mice receiving T lymphocyte infusion was significantly higher than in immunocompetent mice receiving T lymphocyte infusion (control group). As the PD-1^+^ cell frequency declined from day 21 to 35, the total donor cell number moderately increased, indicating substantial loss in PD-1^+^ T cell number.

To examine whether cell death was involved in the process of homeostatic proliferation, CD8^+^ donor cells were stained with BCL-2 and Annexin-V on day 21 after transfer. B-cell lymphoma-2 (BCL-2), an anti-apoptosis protein, could inhibit cell death induced by many cytotoxic factors. The downregulation of its expression indicated cell apoptosis. As shown in **Figures 3B, C**, the expression of BCL-2 significantly decreased while the percentage of apoptosis T cells increased on day 21 after transfer (**Figures 3B, C**). The correlation analysis showed that PD-1 expression on CD8^+^ T cells was significantly negatively related to the killing activity of cytotoxic T lymphocytes and the expression of BCL-2 on CD8^+^ T cells, but positively related to the percentage of apoptosis T cells (**Figure 3D**). These results suggested that T cells transferred to lymphopenic host highly expressed PD-1 and tend to apoptosis on day 21 of homeostatic proliferation.

### Anti-PD-1 antibody treatment enhanced homeostatic proliferation–driven antitumor responses

**Figure 4:**
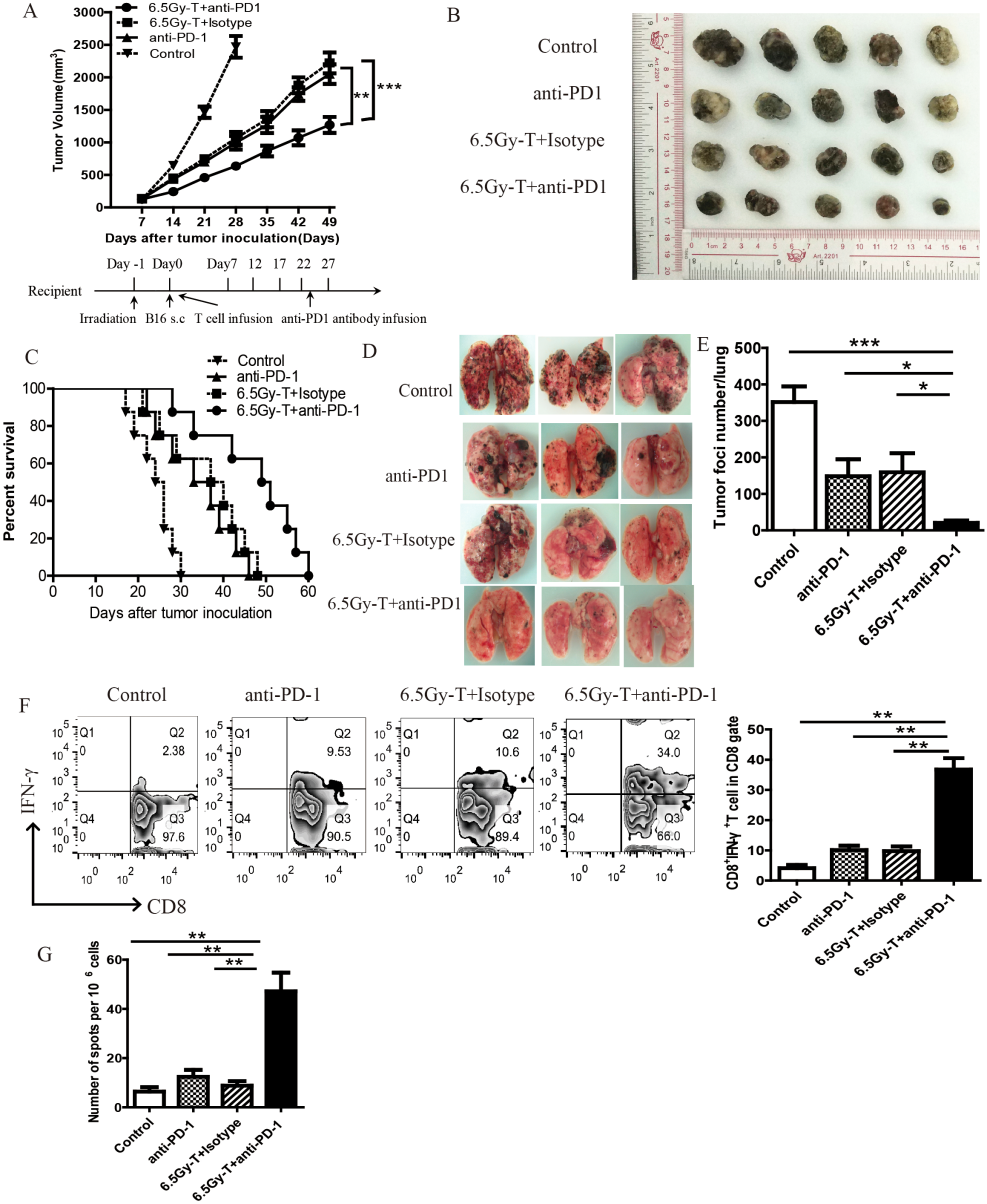
Anti-PD-1 antibody treatment enhanced homeostatic proliferation–driven antitumor responses. **(A)** Tumor growth curve in lymphopenic mice treated with anti-PD-1 antibody after T cell infusion(n=5). Lymphopenic mice induced by total body irradiation treated with anti-PD-1 antibody(6.5Gy-T+ anti-PD-1 groups) or isotype antibody (6.5Gy-T+ Isotype groups) after T cell infusion. Nonirradiated mice were treated with aiti-PD-1 antibody(anti-PD-1 groups) or untreated (Control groups). **(B)** The representative five subcutaneous tumor in each group were shown(n=5). **(C)** Anti-PD-1 antibody treatment significantly inhibited tumor growth and extended mouse survival time in immune reconstitution mice. (n=8, P= 0.000023). **(E)** The number of lung tumor foci in lymphopenic mice treated with anti-PD-1 antibody(n=5). **(D)** The representative three lung tumor was shown. **(F)** Flow cytometry analysis of IFN-γ-releasing CD8^+^ T cells in tumor-DLNs after anti-PD-1 antibody administration(n=5). **(G)** IFN-γ ELISPOT assay were performed in tumor-DLN cells in lymphopenic mice treated with anti-PD-1 antibody(n=5). *P<0.05. **P<0.01. ***P<0.001.

We next evaluated the therapeutic efficacy of anti-PD-1 antibody during reconstitution of the lymphopenic environment. Since PD-1 expression began to rise on day 7 after T cell infusion, we chose this time point to start anti-PD-1 antibody treatment. As shown in **Figure 4**, anti-PD-1 antibody treatment significantly inhibited tumor growth and extended mouse survival time in immune reconstitution mice (**Figure 4A-C**). Moreover, anti-PD-1 antibody treatment significantly reduced the pulmonary metastasis in lymphopenic mice intravenously inoculated with B16 cells (**Figure 4D**).

**Figure 5:**
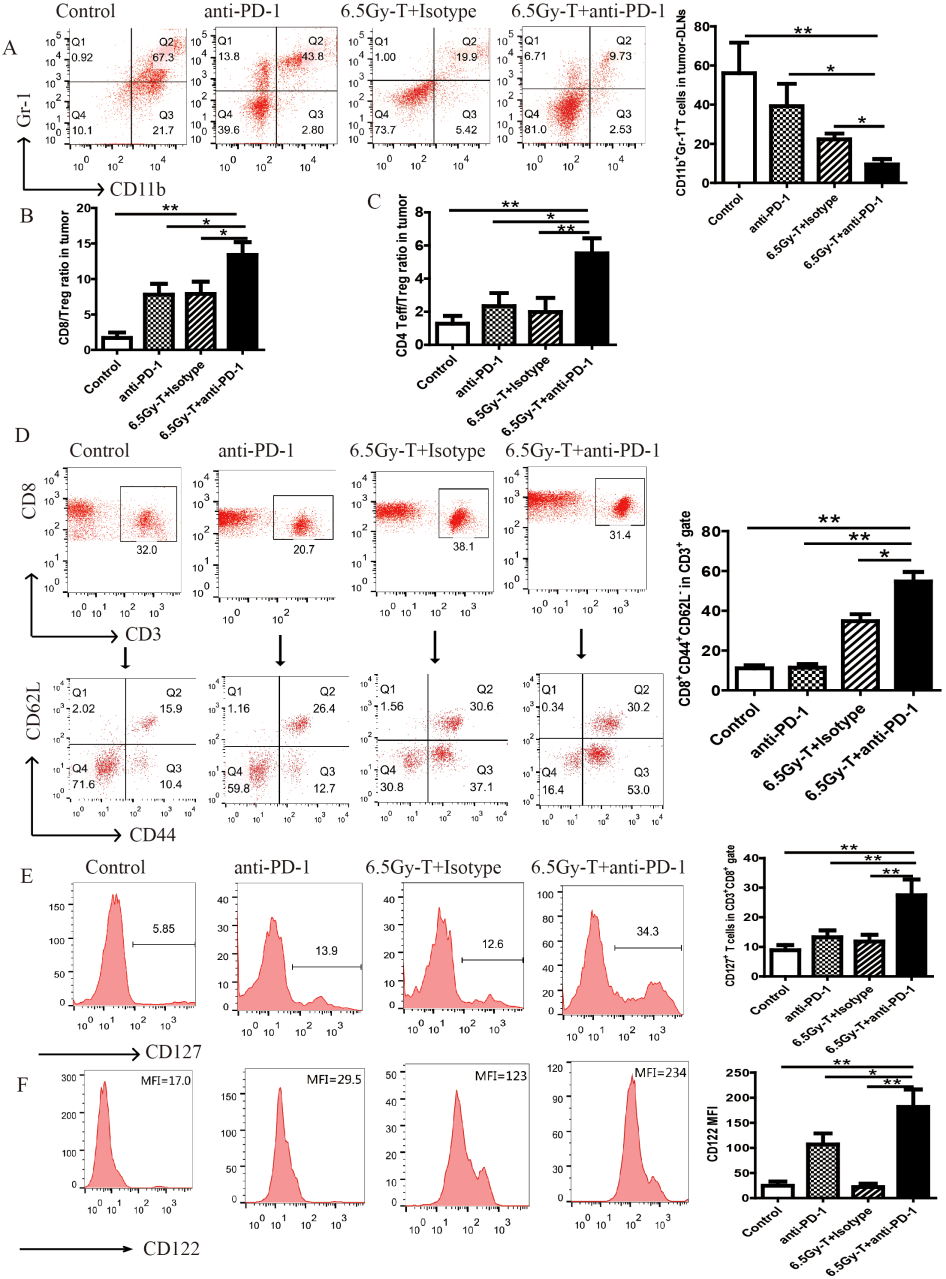
Blocking of PD-1/PD-L1 axis extended the survival of homeostatic proliferation–driven CD8^+^ T cell and promoted it turn into memory T cells. **(A)** Flow cytometry analysis of CD11b^+^Gr-1^+^ MDSCs on CD45^+^ gate in tumor-DLNs(n=5). **(B)** CD8^+^ T cells to Tregs ratios in tumor-DLNs. **(C)** CD4^+^ Effector cells to Tregs ratios in tumor-DLNs(n=5). **(D)** Flow analysis of CD3^+^CD8^+^CD44^+^CD62L^-^ in CD3^+^ gate in tumor-DLNs of lymphopenic mice combined treated with anti-PD-1 antibody and T cell infusion(n=5). **(E)** The ratio of CD127^+^ T cells in CD8^+^ gate in in tumor-DLNs. **(F)** The mean flourscence indensity (MFI) of CD122 in CD8^+^ gate in in tumor-DLNs(n=5). *P<0.05. **P<0.01.

Flow cytometry analysis of IFN-γ-releasing cells and IFN-γELISpot were performed to evaluate the antitumor immunity after anti-PD-1 antibody administration. The percentage of IFN-γ-releasing CD8^+^ T cells on Day21 was significantly increased in lymphopenic mice treated with anti-PD-1 antibody after T cell infusion (**Figures 4F, G**). Regulatory T cells (Tregs) and myeloid-derived suppressor cells (MDSCs), which act as immunosuppressive cell populations, impair antitumor immunity and reduce the clinical efficacy of immunotherapy. To investigate the effects of anti-PD-1 antibody treatment on Tregs and MDSCs during recovery from lymphopenia, we assessed the percentage of MDSCs and Tregs in tumor-DLNs in lymphopenic mice treated with anti-PD-1 antibody after T cell infusion. Anti-PD-1 antibody treatment significantly reduced the recruitment of Tregs and MDSCs in immune-reconstituted mice (**Figure 5A–C**). These data revealed that anti-PD-1 antibody treatment significantly enhanced homeostatic proliferation–driven antitumor responses.

### Blocking of PD-1/PD-L1 axis extended the survival of homeostatic proliferation–driven CD8^+^ T cell and promoted it turn into memory T cells

T cells under homeostatic proliferation usually have two outcomes, apoptosis or turning into memory T cells, at the later stage of homeostatic proliferation. In order to explore whether anti-PD-1 antibody treatment can promote homeostatic proliferation–driven T cells to turn into memory T cells, the ratio of memory T cells were analyzed on day 21 after transfer. CD3^+^CD8^+^CD44^+^CD62L^-^ cells, as effector memory T cells, were significantly increased in lymphopenic mice combined treated with anti-PD-1 antibody and T cells infusion (**Figure 5D**).

Previous studies had demonstrated that IL-7 or IL-15 played an important role on the homeostatic expansion and survival of T cells and indicated the trend of tenting into memory T cells, so we analyzed whether the anti-PD-1 antibody treatment can increase the expression levels of IL-7R (CD127) and IL-15R (CD122) on CD8^+^ T cells. As expected, the expression levels of IL-7R and IL-15R were significantly increased after anti-PD-1 antibody administration (**Figure 5E, F**). It indicated that anti-PD-1 antibody treatment extended the survival of HP cells promoted them turn into memory T cells in the later stage of homeostatic proliferation.

### Blocking of PD-1/PD-L1 axis promoted antigen recognition of HP cells during reconstitution of the lymphopenic environment

**Figure 6:**
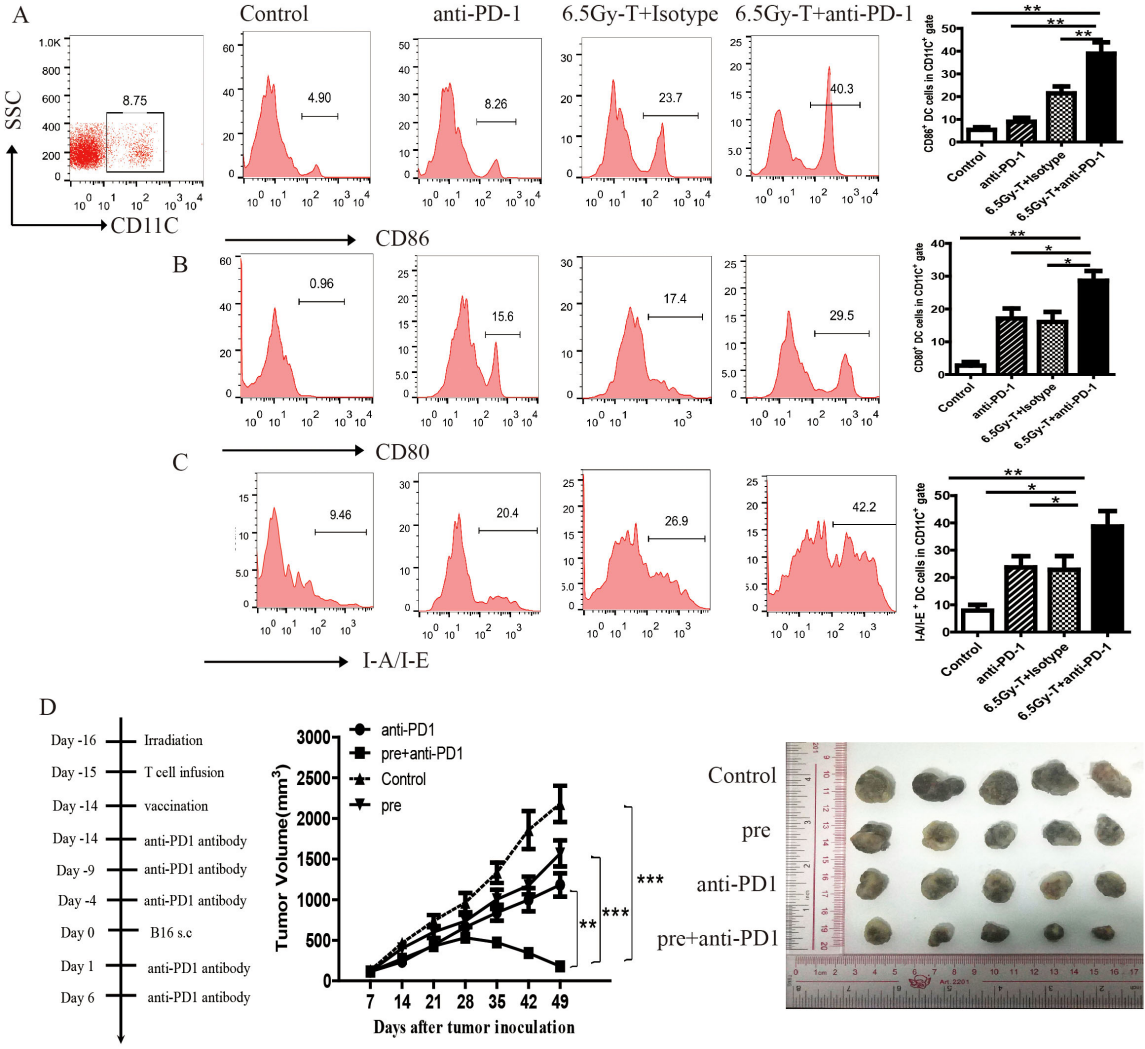
Blocking of PD-1/PD-L1 axis promoted antigen recognition of HP cells during reconstitution of the lymphopenic environment. **(A-C)** The ratio of CD86^+^ **(A)**, CD80^+^ **(B)**, I-A/I-E^+^ **(C)** of DC cells in in tumor-DLNs of lymphopenic mice combined treated with anti-PD-1 antibody and T cell infusion(n=5). **(D)** Tumor growth curve in lymphopenic mice combined treated with irradiated B16 vaccine, anti-PD-1 antibody and T cell infusion. The representative five melanoma tumor was shown(n=5). *P<0.05. **P<0.01. ***P<0.001.

The high ability of TAAs recognition is the important characteristic of HP T cells during the reconstitution of the lymphopenic environment. To evaluate whether blockade of PD-1/PD-L1 axis could enhance the antigen recognition ability of HP cells, functional status of DC cells was assessed using flow cytometry on day 14 after transfer. The co-stimulatory molecules CD80 and CD86 were abundantly expressed on activated dendritic cells, whereas I-A/IE, an MHC-II molecule, was detected on DCs loaded with tumor antigens. As shown in **Figure 6**, the expression of CD80, CD86 and I-A/I-E on DC cells were significantly increased in lymphopenic mice combined treated with anti-PD-1 antibody and T cell infusion when compared with anti-PD-1 antibody or T cell infusion alone (**Figure 6A–C**). These data suggested that blockade of PD-1/PD-L1 could enhance the function of DC cells and promote TAAs recognition during the period of recovery from lymphopenic condition.

A vaccine with TAAs gave lymphopenic mice a tumor antigen for presentation and induce tumor-reactive memory T cells during homeostatic proliferation of T cells. To further assess the effects of anti-PD-1 treatment on TAAs recognition and memory response, we vaccinated lymphopenic mice with s.c. injection of irradiated B16 cells on the day of first anti-PD-1 antibody administration. As depicted in **Figure 6D**, tumor growth was slightly inhibited in vaccinal mice when irradiated B16 cells was s.c. inoculated. Interestingly, anti-PD1 treatment notably enhanced antitumor efficacy and inhibited tumor growth by promoting TAAs recognition and the conversion of memory T cells (**Figure 6D**).

## Discussion

Total body irradiation (TBI) had strong immunomodulatory effects. At lethal dose, TBI was cytotoxic and lymphoablative, causing severe immune suppression and making mice die. At sub-lethal doses, TBI exhibited multifaceted immune-potentiating effects (16). It had been shown that TBI could induce immunogenic tumor cell death that enhanced antigen presentation (17). In addition, immunosuppressive cells, such as Tregs or MDSCs, and anergic immune cells were clear upon irradiation and tumor-induced immune tolerance was broken. More importantly, lymphopenic condition after TBI could create an environment to induce an enhanced antitumor response through the homeostatic proliferation of remaining T cells (18). However, patients undergoing radiotherapy or chemotherapy did not exhibit significantly enhanced antitumor immunity during the recovery period of myelosuppression. It remained undetermined whether either mechanism prevented the antitumor effects triggered by LIP.

In this study, we found that PD-1/PD-L1 play an important role on the reconsitution of immune tolerance against tumor after TBI. We first observed that tumor growth was significantly inhibited when T cells were infused to irradiated mice, and CD8^+^ T cells were the main lymphocyte subsets responsible for the transfer of the antitumor immunity. The lymphopenic condition promoted exogenous or endogenous T cells undergo several rounds of proliferation. In this process, antitumor immunity was driven by the recognition of TAAs and the expansion of tumor reactive T cells.

Despite significant tumor suppression in irradiated mice during the early post-infusion period, this effect was not sustained in mice without anti-PD-1 treatment, because the tumor rapidly regained its growth. Shevyrev et al. also found that antitumor immunity induced by homeostatic proliferation of tumor-specific T cells seem to rapidly decline in association with tumor growth (19). The main reason for failure to completely eliminate the tumor might be the tolerance mechanism of T cells, such as exhaustion and anergy, and this might be influenced by the PD-1/PD-L1 axis. Sustaining antitumor immune responses through anti-PD-1 treatment requires timely administration before immune tolerance develops, specifically in the lymphopenic phase preceding full immune reconstitution.

In this study, we found that despite the continuous proliferation of T cells in tumor-DLNs of lymphopenic mice, the cytotoxicity of tumor reactive CD8^+^ T cells rapidly declined over time. To understand the mechanism of T cell hyporesponsiveness in later stage of T cell homeostatic proliferation, we conducted time course experiments to determine the dynamic change of PD-1 expression on CD8^+^ T cells during recovery from lymphopenic condition. We found that PD-1 expression on CD8^+^ T cells slightly increased on day 7, peaked on day 21, and declined to low levels on day 35. Moreover, the expression of PD-1 was closely related with the apoptosis and hyporesponsiveness of T cells in later stage of T cell homeostatic proliferation.

Anti-PD-1 antibody therapy has demonstrated significant clinical efficacy across various malignancies. However, a subset of cancer patients develops resistance to this treatment modality due to multifactorial mechanisms, including high tumor burden, the presence of an immunosuppressive tumor microenvironment, and concomitant activation of alternative immune checkpoint pathways (20). Exhausted effector T cells could be effectively activated by anti-PD-1 antibody, but they were taken into exhausted state quickly by other immunosuppressive signals in tumor microenvironment (21, 22). In this study, we found that new microenvironment after immune reconstitution can enhance the therapeutic effects of anti-PD-1 antibody treatment. Combined treatment of immune reconstitution and anti-PD-1 antibody significantly inhibited tumor growth and extended mice survival time.

In this study, we found that PD-1/PD-L1 axis played an important role on reformation of immune tolerance in immunologic reconstitution during recovery from lymphopenic condition. There was a synergistic action between anti-PD-1 antibody treatment and immune reconstitution on inhibiting tumor growth and enhancing antitumor immunity. These findings have elucidated the mechanism of the wane of homeostatic proliferation–driven antitumor responses, and provided a new theoretical basis for combined treatment of anti-PD-1 therapy and large dose of radiotherapy or chemotherapy.

## Data Availability

The original contributions presented in the study are included in the article/supplementary material. Further inquiries can be directed to the corresponding authors.
